# Using Twitter to Detect Psychological Characteristics of Self-Identified Persons With Autism Spectrum Disorder: A Feasibility Study

**DOI:** 10.2196/12264

**Published:** 2019-02-12

**Authors:** Yulin Hswen, Anuraag Gopaluni, John S Brownstein, Jared B Hawkins

**Affiliations:** 1 Department of Social and Behavioral Sciences Harvard TH Chan School of Public Health Boston, MA United States; 2 Computational Health Informatics Program Boston Children’s Hospital Boston, MA United States; 3 Department of Mathematics and Statistics Boston University Boston, MA United States; 4 Department of Pediatrics Harvard Medical School Boston, MA United States; 5 Department of Biomedical Informatics Harvard Medical School Boston, MA United States

**Keywords:** autism, digital data, emotion, mobile phone, obsessive-compulsive disorder, social media, textual analysis, tweets, Twitter, infodemiology

## Abstract

**Background:**

More than 3.5 million Americans live with autism spectrum disorder (ASD). Major challenges persist in diagnosing ASD as no medical test exists to diagnose this disorder. Digital phenotyping holds promise to guide in the clinical diagnoses and screening of ASD.

**Objective:**

This study aims to explore the feasibility of using the Web-based social media platform Twitter to detect psychological and behavioral characteristics of self-identified persons with ASD.

**Methods:**

Data from Twitter were retrieved from 152 self-identified users with ASD and 182 randomly selected control users from March 22, 2012 to July 20, 2017. We conducted a between-group comparative textual analysis of tweets about repetitive and obsessive-compulsive behavioral characteristics typically associated with ASD. In addition, common emotional characteristics of persons with ASD, such as fear, paranoia, and anxiety, were examined between groups through textual analysis. Furthermore, we compared the timing of tweets between users with ASD and control users to identify patterns in communication.

**Results:**

Users with ASD posted a significantly higher frequency of tweets related to the specific repetitive behavior of counting compared with control users (*P*<.001). The textual analysis of obsessive-compulsive behavioral characteristics, such as fixate, excessive, and concern, were significantly higher among users with ASD compared with the control group (*P*<.001). In addition, emotional terms related to fear, paranoia, and anxiety were tweeted at a significantly higher rate among users with ASD compared with control users (*P*<.001). Users with ASD posted a smaller proportion of tweets during time intervals of 00:00-05:59 (*P*<.001), 06:00-11:59 (*P*<.001), and 18:00-23.59 (*P*<.001), as well as a greater proportion of tweets from 12:00 to 17:59 (*P*<.001) compared with control users.

**Conclusions:**

Social media may be a valuable resource for observing unique psychological characteristics of self-identified persons with ASD. Collecting and analyzing data from these digital platforms may afford opportunities to identify the characteristics of ASD and assist in the diagnosis or verification of ASD. This study highlights the feasibility of leveraging digital data for gaining new insights into various health conditions.

## Introduction

Autism spectrum disorder (ASD) is a group of developmental disorders that affect >3.5 million Americans [1]. The increasing prevalence of ASD requires greater investigation and methods to diagnose this disorder [2-5]. Diagnosing ASD is difficult, as there is no medical test to diagnose it [6]. Inadequate screening practices [7] and a general lack of awareness of symptoms can lead to delays or misdiagnoses [8]. Symptoms of ASD can sometimes remain unnoticed in childhood as social and language impairments can be subtle and difficult to detect [9].

The Diagnostic and Statistical Manual of Mental Disorders (DSM-5), a widely used classification system, describes ASD as a disorder characterized by marked impairments in social interaction and communication accompanied by a pattern of restricted, repetitive behaviors and activities [10]. However, many of these behaviors may be mistaken for other disorders. For instance, anxiety symptoms may be present in response to social impairments and, as a result, may be considered social anxiety without recognizing the underlying behaviors or the presence of ASD [9]. Reportedly, anxiety is a source and consequence of ASD behaviors and may be a signal to aid in diagnoses [9,11]. In addition, research shows that persons with ASD exhibit higher states of fear and phobia compared with matched patients without ASD [12]. The presence and identification of these symptoms may be markers for persons with ASD coping with judgment faced by social situations [9,13].

Furthermore, common repetitive behaviors observed among persons with ASD may be confused with similarities of having obsessive-compulsive disorder (OCD) [7]. Time-consuming obsessions and compulsions frequently characterize OCD-related behaviors; however, patients with ASD can display repetitive behavior, such as repetitive checking, washing, and counting, which may appear to be compulsive obsessions [14]. Research has found that patients with ASD have significantly fewer compulsions than persons with OCD [15]. Other studies have shown that behaviors like counting, checking, cleaning, and ordering were also displayed less frequently in patients with ASD compared with OCD individuals [16].

Appropriate and timely diagnosis of ASD is necessary to provide proper treatment and symptom management. Numerous diagnostic tools exist today, yet many of these tools face limitations and serve slightly different purposes [17,18]. For example, the Autism Diagnostic Observation Schedule-Generic (ADOS-G) is a widely used diagnostic tool that allows for the inclusion of direct observational data that, for example, the Autism Diagnostic Interview-Revised (ADI-R) lacks [19,20]. However, the ADOS-G often tends to result in the overdiagnosis of ASD [17]. Observational methods and parental interviews have been deemed as helpful to further identify behaviors of ASD, yet these symptoms may often go unnoticed [21,22]. Although multidisciplinary approaches have been considered most effective at producing an accurate diagnosis, these require a significant amount of time, resources, and information, which is not always available [21,23].

Moreover, the use of multidisciplinary approaches extends to different domains, as well as novel sources of information [24]. Recently, exploring digital data has emerged as a promising method for examining human behavior and identifying symptoms of various mental and behavioral conditions [25-27]. Dubbed as the “digital phenotype,” the collection of data from social media, Web-based searches, and use of smartphones and other digital devices can serve as a source of observational information that can yield insights into the lived experiences and everyday realities of persons living with various health conditions [27]. These digital information sources can be further harnessed to potentially detect symptomatic signals to aid in identifying and diagnosing ASD.

Twitter has emerged as a potentially valuable platform for examining a wide variety of behavioral and mental health conditions [25-26,28]. Exploring textual differences in Web-based communication on Twitter is a commonly used method of analyzing affective states and behavioral patterns [29,30]. Previous studies have documented that Web-based data may capture real-time projections of emotional states and health behaviors [26,31]. For example, drawing from analyses of conversations on Twitter, it may be possible to identify the onset of depressive symptoms and detect mood and affective states [32,33]. Given this promising evidence base, there may be opportunities to leverage data from Twitter for identifying the characteristics of ASD.

To date, few studies have used social media for studying ASD, though it may be possible to leverage these popular platforms and novel sources of Web-based data to contribute to a more in-depth understanding of these disorders. A prior study extracted data from Twitter to examine conversations about ASD-related information, thereby demonstrating that this is a topic of frequent discussion on this platform [28]. It may be especially important to consider the use of social media for studying ASD given that a recent report highlighted that roughly 80% of adults living with ASD use popular social media platforms [34]. In this study, our overarching objective is to expand on this prior work and further our understanding about whether publicly available social media data captured from Twitter can yield insights into the presence of digital diagnostic signals for ASD [27,35,36]. In addition, we aim to determine the feasibility of informing a digital phenotype for ASD using social media.

Twitter is a social networking service through which users can post tweets that can contain up to 140 characters (increased to 280 characters in November 2017). Twitter is a prominent social medium, as evidenced by its >336 million monthly active users [37]. Twitter is a source of observational data that can yield rich data, including real-time thoughts, feelings, and attitudes, which traditional surveys are typically unable to capture. Therefore, this study aims to compare the textual patterns of communication of Twitter users who self-identify as having ASD to a general population of control users to identify textual signals related to fear, anxiety, and paranoia, which have often been recognized as a response to symptoms of ASD among individuals living with the disorder [12]. Investigating discussion surrounding these emotions for Twitter users with ASD can potentially enable the detection of Web-based emotional signals that can provide more information to better inform diagnoses of ASD. In addition, this study aims to explore common OCD behaviors using the Yale-Brown Obsessive-Compulsive Scale (Y-BOCS) to understand whether these symptoms are present among Twitter users with ASD and whether the presence or absence of digital obsessive-compulsive symptoms could assist in reducing misdiagnoses of ASD. Finally, we investigated the timing of social media communications to explore differences in patterns of social communication between Twitter users with ASD and control users. Information collected from Twitter as a novel digital data source could potentially guide and assist in the clinical diagnoses and treatment of ASD.

## Methods

### Data Collection

In this study, we retrieved data from the Twitter application programming interface and collected meta-data such as Twitter users’ total number of tweets, number of followers, number of friends, and favorites. We collected data from Twitter between March 22, 2012 and July 20, 2017. The trajectories of ASD symptoms are not stable and can fluctuate over the course of a few months or even years, especially during developmental periods. A 5-year period was selected to ensure that symptoms would be detected even with fluctuations to capture these detectable differences between the 2 groups. This study was considered exempt from ethical review because only publicly available Web-based data collected from the Twitter platform were analyzed. We acknowledge the ethics of drawing in data from users who self-identify as ASD. To ensure the privacy of data from this group, data collected were aggregated and analyzed at the population level and were anonymous in this format. Furthermore, this study only used data from users who consented on Twitter to disclose their data publicly (ie, no privacy settings were selected by users) and are completely public.

### Twitter Users

We identified the cohort of Twitter users with ASD by self-identification and explicit Twitter discussion of having ASD. If users described or labeled themselves as autistic or having ASD, then they were determined to be Twitter users with ASD. We searched for terms relating to ASD in users’ tweets, and users were considered Twitter users with ASD if they tweeted or wrote a caption about their condition or diagnosis of ASD. We used a previously validated approach to recruit participants with behavioral disorders by Hswen et al [25]. We retrieved tweets that contained one or more keywords associated with ASD; these keywords were searched for in the content of users’ tweets and information in their Twitter profile. The keyword list is as follows: autism, autistic, Spectrum Disorders, #ASD, #ActuallyAutistic, #AutismSpeaks, #AutismSpeaks10, #AutismParent, #Autchat, #AutismAwareness, #LIUB, aspergers, and aspies. To validate if users did have ASD, 2 independent researchers coded users if they had ASD or not and an agreement from 2 researchers was necessary for a user to be labeled as a person with ASD. If a disagreement occurred, a third researcher was used to tie break the labeling of the user. To the best of our knowledge, this is the first study to identify ASD users through Twitter, and it is an exploratory study for the identification and labeling of ASD users by machine learning classification and manual curation. A random sample was selected that did not have a self-identified user caption or tweet mentioning ASD and served as the control group. Originally, 220 users with ASD and 220 control users were selected. After filtering out bots and spam accounts and inaccessible accounts (ie, accounts that were private, deleted, deactivated, or banned), we enrolled 152 users with ASD and 182 control users for analysis.

### Textual Analysis of Tweets Related to Fear, Anxiety, and Paranoia

We examined the tweets related to the emotions of fear, anxiety, and paranoia through textual analysis. These emotions were selected on the basis of previous research studies that have identified these emotions as key differentiating emotions of persons with ASD compared with matched populations without an ASD diagnosis [12]. We aimed to collect all discussion about these 3 emotional states. The root keyword of the emotion was expanded to all tenses and pluralities to capture the breath of tweets related to that emotion (Table 1). Manual selection of these terms was used to ensure that users were explicitly referring to the emotion in their conversations, a procedure we have applied in prior studies [25]. In addition, the terms “scare” and “scared” were used in the category of fear as a qualitative exploration of discussions around fear to express fearful states. Tweets from users with ASD and control users were queried for all tweets that contained keywords for each category. The number of tweets that contained each keyword was summed to create the total number of tweets for each emotion category. A tweet that contained instances of multiple words, such as “anxious” or “anxiety,” was counted as 1 tweet. Overlapping keyword categories, such as a tweet that contained “anxious” and “fear,” were counted as 1 tweet per category.

### Tweets Containing Obsessive-Compulsive Disorder–Related Keywords

To examine the presence of OCD-related tweets, we compiled a list of key terms from symptom categories within the Y-BOCS. First developed by Goodman et al, this widely used checklist comprehensively covers significant OCD-related behaviors [38]. From the Y-BOCS, 13 categories were selected on the basis of previous research that identified these specific behaviors to be confused with symptoms of OCD and can be mislabeled as an OCD diagnosis [14-16]. These OCD symptom categories included the following: obsess; fixate; repeat; routine; freak; clean; check; count; hoard; wash; worry; excess; and concern. Stem words were expanded with the same methodology as applied for emotion keywords to conduct textual analysis (Table 2). For each category, the number of tweets containing these terms was collected and summed to generate the total number of tweets per OCD category.

**Table 1:** All terms related to fear, anxiety, and paranoia.

Root keyword	All forms of keyword
fear	fear, feared, fearful, fearing, fears, scare, and scared
anxiety	anxieties, anxiety, anxious, and anxiousness
paranoia	paranoia, paranoiac, paranoiacs, and paranoid

**Table 2:** All terms related to obsessive-compulsive disorder–related keywords.

Root keyword	All forms of keyword
obsess	obsessed, obsessing, obsession, obsessions, obsessive, obsessively
fixate	fixated, fixation, fixations
repeat	repeat, repeated, repeatedly, repeating, repeats, repetition, repetitions
routine	routinely, routines
freak	freaked, freaking, freakout, freakouts
clean	cleaned, cleaning
check	checking, checked, checks, recheck, rechecks, rechecked, rechecking
count	counted, counting, counts, recount, recounted, recounting, recounts
hoard	hoarded, hoarder, hoarders, hoards
wash	washed, washing, washes
worry	worried, worrying
excess	excessive, excessively, excessiveness
concern	concerned, concerning, concern

### Mean of Proportions for Each Category

As a method to standardize the variation of the frequency of tweets for each user, a “mean of proportions” was calculated for each category. This measure considers the mean number of tweets by category and the proportion of users that tweeted the keywords in each category for emotions and OCD terms. For each user, the total number of tweets for each category was divided by the users’ total number of tweets during the full study period between March 22, 2012 and July 20, 2017 (a total of 1946 days). This measure aimed to control the volume of tweets by users to ensure that the mean number of tweets by category was not skewed by individual users’ frequency of tweets. The mean of each proportion for users with ASD and the control user group was calculated by dividing the proportion of tweets by category over the number of users within each group. Of note, we used these means of proportions values solely for comparison between the ASD user group and the control user group; we do not know or study the clinical significance of the values.

### Timing of Tweets

To examine differences in social communication patterns between users with ASD and control users, we analyzed the timing of tweets between the 2 groups to identify whether tweets occurred at different times of the day between the 2 groups. Meta-data were collected on the local time of users’ tweets using the universal time code (UTC) offset data. Twitter users can choose to select whether to include their local time zone in their account settings, and tweets with UTC offset data were included in this analysis. These tweets were further classified into the following time intervals: 00:00-05:59; 06:00-11:59; 12:00-17:59; and 18:00-23:59.

### Statistical Analyses

We conducted 2-tailed Welch’s *t* tests to compare the “mean of proportions” for each category of emotion and OCD keywords between users with ASD and control users. A chi-square test was used to compare the proportion of users in each category of the timing of tweet data between the 2 groups. For all tests, a *P*<.05 was considered statistically significant. All analyses were performed with the Python Programming Language Version 3.6 (Python Software Foundation).

## Results

### Characteristics of Twitter Users

In total, 4,093,559 tweets were collected between March 22, 2012 and July 20, 2017. Twitter users with ASD (n=152) tweeted a total of 1,700,841 tweets, and control users (n=182) tweeted a total of 2,392,718 tweets. We observed no statistically significant differences between the group of users with ASD and the group of control users in the mean number of tweets per user or for meta-data, including the number of friends, followers, or favorites (Table 3).

### Mean Proportion of Tweets for Fear, Anxiety, and Paranoia

**Figure 1:**
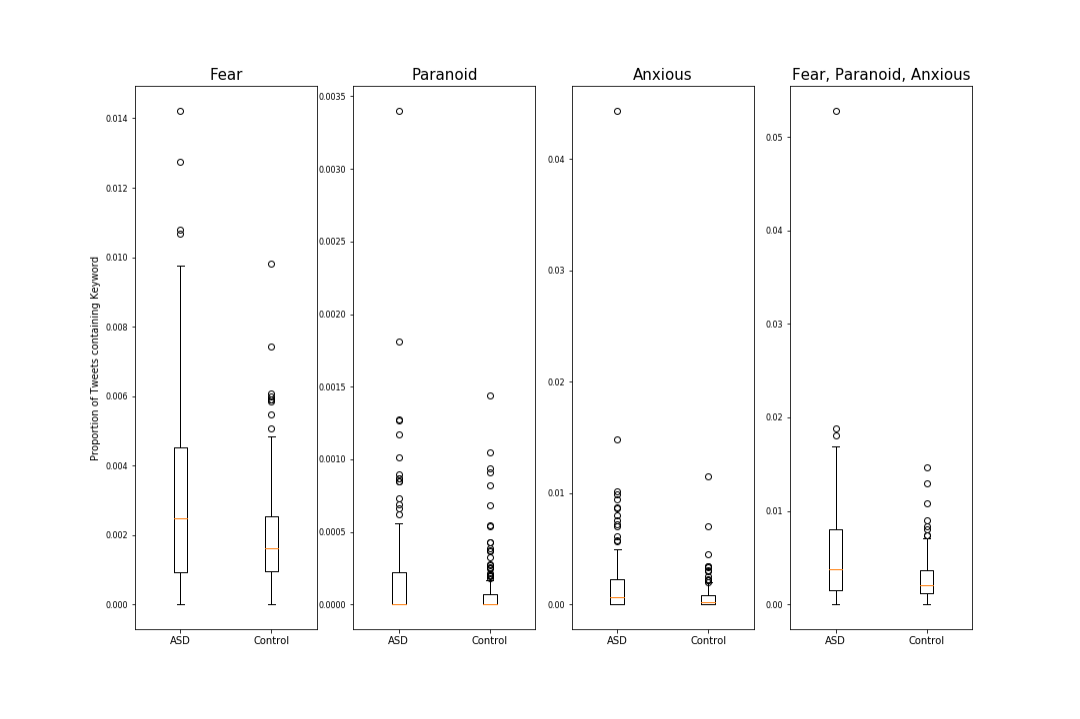
Differences in emotions between autism spectrum disorder (ASD) and control users.

Users with ASD posted a greater number of tweets compared with control users for all 3 emotions—fear (3.11e-03 vs 1.98e-03; *t*_225_=4.410, *P*<.001), anxiety (1.91e-03 vs 6.49e-04; *t*_172_=3.529, *P*=.001), and paranoia (1.90e-03 vs 8.12e-05; *t*_211_=3.021, *P*=.003; Table 4; Figure 1). Aggregating all 3 emotions together, Twitter users with ASD posted a higher number of tweets compared with control users (5.15e-03 vs 2.68e-03; *t*_190_=4.981, *P*<.001; Table 4; Figure 1).

### Mean Proportion of Tweets for Obsessive-Compulsive Disorder–Related Terms

**Figure 2:**
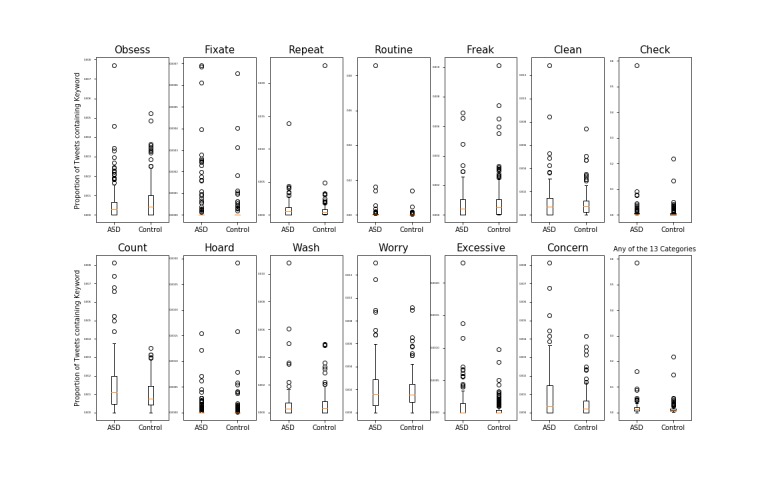
Differences in obsessive-compulsive disorder-related discussion between autism spectrum disorder (ASD) and control users.

Users with ASD posted a higher number of tweets compared with control users for 4 OCD-related keyword categories—fixate (3.64e-05 vs 1.23e-0.5; *t*_230_=2.356, *P=*.02), count (1.43e-03 vs 9.69e-04; *t*_219_=3.457, *P*<.001), excessive (1.15e-04 vs 4.82e-05; *t*_201_=2.807, *P*=.005), and concern (9.26e-04 vs 4.55e-04; *t*_219_=3.959, *P*<.001; Table 5; Figure 2). No statistically significant differences between users with ASD and control users were observed for the remaining 8 OCD-related categories (Table 5; Figure 2). In addition, upon aggregating all OCD-related terms, no statistically significant difference between groups was observed (1.20e-02 vs 1.44e-02; *t*_193_=1.292, *P*=.20; Table 5; Figure 2).

### Timing of Tweets

The number of Twitter users who had UTC offset data necessary to retrieve the timing of tweets was 69.2% (231/334). We observed no statistically significant difference in the availability of time zone information between users with ASD (111/152, 73.0%) compared with control users (120/182, 65.9%; *χ*^2^_1_=2.0, *P*=.16). A lower proportion of tweets from users with ASD compared with control users was observed during the time intervals of 00:00-05:59 (222,810/1,700,841, 13.10% vs 330,915/2,392,718, 13.83%; *P*<.001), 06:00-11:59 (294,245/1,700,841, 17.30% vs 468,972/2,392,718, 19.60%; *P*<.001), and 18:00-23:59 (612,302/1,700,841, 36.00% vs 890,091/2,392,718, 37.20%; *P*<.001; Table 6). A higher proportion of tweets from users with ASD compared with control users was observed during the time interval 12:00-17:59 (571,483/1,700,841, 33.60% vs 703,459/2,392,718, 29.40%; *P*<.001; Table 6).

**Table 3:** The characteristics of Twitter users with autism spectrum disorder (ASD) and control users.

Twitter user characteristics	Twitter users with ASD (n=152), mean (SD)	Control twitter users (n=182), mean (SD)	*t* (*df*)^a^	*P* value
Mean overall tweets per user	11,189 (23,019)	13,146 (20,159)	–0.818 (303)	.41
Mean retweets per user	2237 (4886)	4942 (9680)	–3.299 (277)	.001
Mean original tweets per user	8952 (20,663)	8204 (12,369)	0.391 (237)	.70
Number of friends	1460 (5422)	1193 (5559)	0.442 (324)	.66
Number of followers	1778 (5753)	1891 (7752)	0.153 (327)	.88
Number of favorites	15,556 (31,966)	12,515 (19,978)	1.018 (243)	.31

^a^2-tailed Welch’s *t* tests were used to compare the “mean of proportions” for each category.

**Table 4:** Tweets containing emotion-related keywords among Twitter users with autism spectrum disorder (ASD) and control users.

Emotion terms category	Twitter users with ASD (n=152), mean (SD)	Control twitter users (n=182), mean (SD)	*t* (*df*)^a^	*P* value
fear	3.105e-03 (2.822e-03)	1.975e-03 (1.553e-03)	4.410 (225)	<.001
paranoid	1.902e-04 (4.057e-04)	8.120e-05 (1.993e-04)	3.021 (211)	.003
anxious	1.914e-03 (4.275e-03)	6.486e-04 (1.230e-03)	3.529 (172)	.001
Tweets with any of the 3 emotional categories’ terms	5.154e-03 (5.760e-03)	2.679e-03 (2.282e-03)	4.981 (190)	<.001

^a^2-tailed Welch’s *t* tests were used to compare the “mean of proportions” for each category.

**Table 5:** Tweets containing obsessive-compulsive disorder (OCD)–related keywords among Twitter users with autism spectrum disorder (ASD) and control users.

OCD keyword category	Twitter users with ASD (n=152), mean (SD)	Control twitter users (n=182), mean (SD)	*t* (*df*)^a^	*P* value
obsess	5.826e-04 (9.807e-04)	7.086e-04 (9.377e-04)	–1.193 (316)	.23
fixate	3.640e-05 (1.120e-04)	1.226e-05 (6.404e-05)	2.356 (230)	.02
repeat	8.711e-04 (1.381e-03)	7.239e-04 (1.775e-03)	0.852 (330)	.40
routine	1.027e-03 (7.171e-03)	2.866e-04 (1.114e-03)	1.260 (157)	.21
freak	7.584e-04 (1.066e-03)	8.827e-04 (1.342e-03)	–0.943 (331)	.35
clean	1.068e-03 (1.534e-03)	9.481e-04 (1.026e-03)	0.821 (255)	.41
check	1.061e-02 (4.840e-02)	6.944e-03 (1.982e-02)	0.874 (193)	.38
count	1.427e-03 (1.471e-03)	9.693e-04 (7.744e-04)	3.457 (219)	.001
hoard	5.608e-05 (1.876e-04)	4.938e-05 (2.616e-04)	0.272 (325)	.79
wash	5.766e-04 (1.176e-03)	5.986e-04 (8.416e-04)	–0.193 (267)	.85
worry	2.071e-03 (2.167e-03)	1.878e-03 (1.454e-03)	0.934 (256)	.35
excessive	1.152e-04 (2.728e-04)	4.816e-05 (1.215e-04)	2.807 (201)	.005
concern	9.260e-04 (1.322e-03)	4.548e-04 (6.964e-04)	3.959 (219)	<.001
Tweets with any of the 13 categories’ terms	1.997e-02 (4.940e-02)	1.445e-02 (2.023e-02)	1.292 (193)	.20

^a^2-tailed Welch’s *t* tests were used to compare the “mean of proportions” for each category.

**Table 6:** Timing of users’ tweets between Twitter users with autism spectrum disorder (ASD) and control users.

Time interval	Proportion of tweets (%)		*χ*^2^ (*df*)	*P* value
	Among twitter users with ASD	Among control twitter users		
00:00-05:59	13.1	13.8	351.9 (*1*)	<.001
06:00-11:59	17.3	19.6	2952.9 (*1*)	<.001
12:00-17:59	33.6	29.4	6713.7 (*1*)	<.001
18:00-23:59	36.0	37.2	474.3 (*1*)	<.001

## Discussion

### Principal Findings

The diagnosis of ASD is complex. Often a multidisciplinary approach is required to produce the most accurate diagnosis. This study investigated the feasibility of using Twitter as a novel digital platform for generating additional insights to assist in the diagnosis of ASD. The findings revealed that through the textual analysis of tweets, discussions about commonly heightened emotional states associated with ASD are expressed among Twitter users who self-identify as having ASD. Specifically, Twitter users with ASD tweeted more frequently about fear, anxiety, and paranoia compared with a randomly generated control group of Twitter users. In addition, these results suggest that Web-based emotional patterns expressed among Twitter users with ASD may parallel offline emotional symptoms known to be associated with the condition and, therefore, may be used as a signal for detecting or confirming a diagnosis of ASD.

As misdiagnoses of ASD for OCD are common because of many overlapping and similar symptoms [39,40], in this study, we also explored whether there may be differences in the expression of OCD-related behaviors among Twitter users with ASD compared with control users. Of 13 selected OCD-related categories, users with ASD discussed only 4 categories—fixate, count, excessive, and concern—significantly more than the control user group. Furthermore, when aggregating all 13 categories, we observed no statistically significant difference. These findings indicate that Twitter users with ASD may not display a wide number of symptoms of OCD and may highlight the most salient symptoms that could be explored further as a method to distinguish symptoms of ASD from individuals with OCD.

Although research on the presence of some of these behaviors may steer clinicians toward a diagnosis of OCD, misattribution to a behavior or misdiagnosis can lead to the inappropriate treatment for ASD [7,41]. Therefore, it is important to consider the spectrum of symptoms of OCD and determine whether Web-based discussions of these behaviors are consistent with offline representations of OCD. However, this highlights an important area for future investigation because offline observational information of this variety of symptoms can be difficult to obtain. Ideally, there may be opportunities to leverage Web-based social media data that allow us to capture minute-by-minute thoughts and behaviors to provide clinicians a more objective illustration of patients’ behaviors toward better understanding the daily occurrences of these symptoms and inform diagnosis and treatment recommendations.

Finally, we sought to determine differences in patterns of the timing of Twitter communication between users with ASD and control users. As ASD is characterized as a social disorder [41], there could be differences in patterns of the Web-based communication timing among individuals with this condition compared with other mental disorders. We found some differences in the proportion of tweets between groups at each time-point throughout the day; however, these differences were very small and are likely not indicative of any diagnostically relevant differences between groups. This is consistent with previous studies that have found no differences in the timing of tweets between a general population of Twitters users and users with mental disorders such as schizophrenia [25]. Additional research is needed to explore whether potential differences in the timing of social media use and posts between users with ASD and control users could inform the development of targeted clinical and public health interventions to reach this patient group online.

### Limitations

Although Web-based social media offers an opportunity to observe a wide range of thoughts and behaviors, our data have some limitations. First, Twitter users who self-identify as having ASD may not be representative of the general population of individuals with ASD. The Twitter population, on average, is composed of a larger proportion of younger adults aged 18-29 years with higher levels of education compared with those who do not use Twitter [42]. Therefore, it is more likely that users with ASD in our study are younger adults and with higher education levels than persons with ASD who do not use social media. Second, identification of users with ASD was through self-identification on Twitter. Many individuals with ASD who are on Twitter may not openly self-identify as having ASD, thereby making the sample included in this study potentially more comfortable discussing their disorder over the Web with others. This might mean that our study population is different compared with Twitter users with ASD who choose not to self-identify as having ASD online. Although no formal clinical diagnosis was confirmed for Twitter users included in the analyses conducted in this study, the nature of the stigmatizing diagnosis of ASD makes it highly unlikely that individuals would be dishonest about publicly self-identifying as having ASD on Twitter. There is a possibility, however, that there are some who might have self-diagnosed themselves as having ASD without a clinical consultation, and we, therefore, cannot verify the clinical diagnosis of ASD in our study population. Of note, this was an exploratory study that used textual analyses to explore whether common emotions and OCD-related behaviors could be detected among Twitter users with ASD and whether these discussions may differ compared with control users. As a result, we can only report on publicly available discussions captured from Twitter and, therefore, we are unable to confirm whether these are also occurring offline. In addition, we did not identify a cohort of OCD users and did not conduct a comparison between ASD and OCD users. Therefore, we are not able to confirm the differences in Web-based patterns between the 2 groups and further examination is necessary. Furthermore, we note that we developed an adaptive list for the emotion categories related to autism but did not expand this list for the OCD-related categories. We specifically used the terminology used in the Y-BOCS to mimic the survey and the terms used to diagnose in the clinical setting. However, we acknowledge that these OCD terms could have been adapted; therefore, we potentially missed OCD-related behaviors in our analysis of tweets. Finally, we acknowledge that small yet detectable differences were yielded from our analyses. Owing to the size and breadth of our dataset, although small, these differences can detect important differences in the characteristics between ASD users and the general population and are worth exploring further as to what is a meaningful change to influence a diagnosis.

The strength of this study is that this is the first known study to leverage Web-based social media data from Twitter to compare emotional and OCD-related discussions between users with ASD and a randomly select group of control users. A comprehensive picture of a digital footprint of emotions and behaviors can be gleaned from Twitter through the examination of Web-based social media data. This study highlights the feasibility of conducting this type of analysis through these emerging digital streams to further contribute to our understanding of a “digital phenotype” for ASD. This study yields additional insights that contribute to a growing body of evidence depicting digital phenotyping and the potential to draw from Web-based data sources to detect signals and symptoms for a range of disorders such as ASD [27,35,36]. The data captured in this way were in a naturalistic and unsolicited format, thereby avoiding the concerns of social desirability bias or recall bias that are present with traditional survey data collection methods. Therefore, the methods applied in this study highlight the potential of collecting novel data streams that could be used to supplement the existing data collection methods. Furthermore, it is important to note that we are dealing with individuals from vulnerable population groups, and they are contributing their conversations and thoughts for observational research studies such as ours; however, all of these publicly available data are always aggregated, and we purposefully did not identify the individuals.

### Conclusions

Digital signals from Web-based social media may offer an additional resource to capture information not always available for direct observation. This Web-based data source is not meant to replace traditional forms of diagnosis but to supplement these existing approaches by helping to identify symptoms or confirm diagnoses of ASD. Multidisciplinary approaches are often required to gain a full breadth of information and appear to be the gold standard of diagnostic methods. The accuracy of diagnoses is necessary to ensure that persons with ASD receive proper treatment and care in a timely manner so that they can live a fulfilling and healthy life [21,43-45]. Findings from this study can contribute to the development of multidisciplinary clinical assessments that draw from novel digital sources, as well as traditional surveys and clinical interviews, and takes steps toward illustrating the potential feasibility of digital detection methods for ASD.

## Abbreviations

ASD: autism spectrum disorder
OCD: obsessive-compulsive disorder
UTC: universal time code
Y-BOCS: Yale-Brown Obsessive-Compulsive Scale
